# Past, Present and Future of Research on Wearable Technologies for Healthcare: A Bibliometric Analysis Using Scopus

**DOI:** 10.3390/s22228599

**Published:** 2022-11-08

**Authors:** Yolanda-María de-la-Fuente-Robles, Adrián-Jesús Ricoy-Cano, Antonio-Pedro Albín-Rodríguez, José Luis López-Ruiz, Macarena Espinilla-Estévez

**Affiliations:** 1 Social Work Department, University of Jaén, 23071 Jaén, Spain; 2 Education and Sports Council, Junta de Andalucía (Regional Government of Andalusia), 23007 Jaén, Spain; 3 Computer Science Department, University of Jaén, 23071 Jaén, Spain

**Keywords:** wearable technologies, healthcare, wearable sensors, bibliometric analysis, Sustainable Development Goals, VOSviewer, CiteSpace, scientific visualization

## Abstract

Currently, wearable technology is present in different fields that aim to satisfy our needs in daily life, including the improvement of our health in general, the monitoring of patient health, ensuring the safety of people in the workplace or supporting athlete training. The objective of this bibliometric analysis is to examine and map the scientific advances in wearable technologies in healthcare, as well as to identify future challenges within this field and put forward some proposals to address them. In order to achieve this objective, a search of the most recent related literature was carried out in the Scopus database. Our results show that the research can be divided into two periods: before 2013, it focused on design and development of sensors and wearable systems from an engineering perspective and, since 2013, it has focused on the application of this technology to monitoring health and well-being in general, and in alignment with the Sustainable Development Goals wherever feasible. Our results reveal that the United States has been the country with the highest publication rates, with 208 articles (34.7%). The University of California, Los Angeles, is the institution with the most studies on this topic, 19 (3.1%). Sensors journal (Switzerland) is the platform with the most studies on the subject, 51 (8.5%), and has one of the highest citation rates, 1461. We put forward an analysis of keywords and, more specifically, a pennant chart to illustrate the trends in this field of research, prioritizing the area of data collection through wearable sensors, smart clothing and other forms of discrete collection of physiological data.

## 1. Introduction

Currently, electronic health (eHealth) is understood as an emerging field of medical informatics, referring to the organization and provision of health services and information using the Internet and related technologies for the improvement of healthcare at all levels [1]. eHealth, made up of the set of Information and Communication Technologies (ICTs) that are used for prevention, diagnosis, treatment, monitoring and health management, is considered one of the fastest growing areas in health, saving costs in healthcare systems and improving effectiveness and efficiency [2]. Since 2016, with the World Health Organization’s EB 142/20 report, the WHO’s executive board has considered the use of mobile health (mHealth) to be of vital importance—that is, mobile wireless technologies for public health, specifically in health monitoring. This report declares the usefulness of mHealth in increasing access to health information, services and skills, and it also makes clear how these tools promote positive changes in health behavior and disease control [3].

Subsequently, in 2018, the WHO expanded the range of coverage of mHealth to include the use of other digital technologies for public health. Resolution WHA71.7 urged member states to prioritize the design and use of digital technologies as a mechanism for promoting Universal Health Coverage and the Sustainable Development Goals [4,5].

This type of technology is perfectly aligned with Sustainable Development Goal (SDG) 3 (Health and Well-being). Currently, there are a multitude of wearable devices capable of measuring physiological data such as heart rate, steps, blood oxygen levels or even electrocardiograms. All these data can be used to analyze the health status of the user and improve their well-being, allowing synergy with SDG 3.

An increasing number of these types of devices are designed in a sustainable way by promoting repair, mending or recycling, the use of the collaborative economy, ensuring the second life of products or contributing to a circular wearable device system. In this case, it is aligned with SDG 11 (sustainable cities and communities) and SDG 12 (responsible production and consumption).

In this way, notable progress is observed from initiatives such as the European Commission’s eHealth Action Plan 2012–2020. Although it already included a special approach to mHealth, this Plan was more focused on offering patients and healthcare workers better access to the most recent initiatives mentioned above [6] in connection to other types of information technologies and devices (eHealth) in the healthcare system.

These developments are emerging against the backdrop of population aging, which can be described as a global phenomenon. Every country in the world is experiencing an exponential growth in the proportion of older people. While in 2019 the figures indicated that there were around 703 million people aged 65 or over in the world, it is expected that by 2050 the number of elderly people will double, reaching 1500 million [7]. Wearable technologies (WT) make up a set of devices that are an integral part of providing solutions in healthcare to a world population characterized by notable demographic aging [8].

Recently, trends in research have broadened their focus, and numerous studies have been conducted evaluating the impact of WT in different populations and with different objectives. Thus, depending on their role, we can find studies on WT aimed at preventing diseases and injuries [9,10,11,12,13]; diagnostic support systems [14,15,16,17]; rehabilitation [18,19,20,21,22]; and mainly for health surveillance and monitoring [23,24]. 

Currently, the importance of having precise control of physiological measurements, such as heart rate variability and heart beats per minute, has become clear. Abnormal changes in these indicators can be an early sign of respiratory infections such as those caused by the SARS-CoV-2 virus (COVID-19) [25]. This information can be easily collected by wearable wrist biometric devices and smartphones [26].

Likewise, there is ever more research on the applications of wearable devices as support systems in detection, diagnosis, acquisition and processing of data related to disorders and chronic conditions in populations with different age ranges [27,28,29,30]. 

Similarly, electrochemical sensors and wearable biosensors are becoming increasingly important in the field of non-invasive or minimally invasive monitoring of the health status of individuals. This may be mainly due to certain specific characteristics such as their performance, inherent miniaturization, low cost and wide applicability [31]. In particular, wearable chemical sensors offer the opportunity to monitor (bio)chemical parameters through body biofluids. Another advantage of these systems is the possibility of directly converting (bio)chemical information to the digital domain, which facilitates the interpretation of data by professionals from different areas [32]. Biological fluids provide clinically useful information on the health status of individuals. In these non-invasive or minimally invasive samples, body fluids that are easily accessible, such as saliva, sweat, blood or urine, have been used [33,34].

These data on biochemical activity, collected by discrete and long-term monitoring, will be essential for the control of many chronic diseases such as diabetes, gout, or Parkinson’s disease [35].

Due to this significant increase in scientific production in the field of wearable technology in healthcare (WTH), it seems relevant to carry out a bibliometric study with the aim of outlining the evolution of the intellectual structure of this area of knowledge over time. Our goal is to analyze and map scientific advancements in WTH, identify future challenges within the field and put forward some proposals to address them. More specifically, this paper tries to answer the following research questions:

- General Research Question: How has the scientific production published in Scopus within the research field of wearable technologies for health monitoring evolved over the last two decades?
  - Specific Research Question (SRQ) 1: How many specific publications are there on this subject in Scopus and what trends can be observed?
  - SRQ 2: What countries and institutions produce most of this research?
  - SRQ 3: Which are the most active journals based on the objectives of this research and the relationship between them?
  - SRQ 4: Who are the most relevant authors based on the search strategy proposed in this analysis and the co-citation between them?
  - SRQ 5: What are the most significant key concepts and how have they evolved over the years?

Among the innovations for carrying out this bibliometric study are: (1) to offer precise bibliometric information that allows researchers to make informed decisions in relation to their research, such as: which works they could cite, in which journals to publish and to identify authors or institutions of relevance in the field with which they can collaborate; (2) identify new lines and areas of research, determine the obsolescence of certain topics and the performance of scientific activity; (3) identify possibilities, limitations and challenges that portable devices face in data processing; (4) underline the importance of collaboration between researchers, manufacturers and healthcare providers to understand the needs of the market and to be able to meet critical health and safety requirements in the design and production of sensors.

This manuscript is structured as follows: Section 1 presents the subject matter of our work, introducing the contributions of WT in relation to public health. Section 2 presents a detailed review of previous research and its contributions to this knowledge area. Section 3 explains the proposed methodology for the detection and selection of studies, and their subsequent analysis in detail. Section 4 presents the main results obtained in our bibliometric analysis; it begins with a description of the studies’ achievements and proceeds with a mapping of the papers included in the analysis. In Section 5, the results in previous research are discussed and the implications and limitations of our study are presented. Finally, in Section 6, our conclusions will be drawn, pointing to future challenges and putting forward some proposals.

## 2. Literature Review

### 2.1. Definition, Characteristics and Opportunities of Wearable Technologies

“Wearable technology” (WT) or “wearable devices” are concepts used to refer to electronics and computers that are integrated into garments, as well as other devices and accessories that can be worn comfortably on our body [36]. The rapid advances in wearable devices had already been anticipated in a 2016 review of WT in medicine [37]. In the aforementioned study, a total of 13 different body sensors and 11 head-mounted display devices were analyzed by means of a bibliographic review. The latter stood out mainly for their use in surgery, quality imaging, simulations for educational purposes and as navigation tools, while body sensors were used to monitor vital signs, postural control and physical status.

Han, et al. (2014) [38] present a list of the characteristics of wearable smart devices:

(1) Wearable smart devices should not significantly change people’s habits or usual way of life while they are in use.
(2) These devices perform specific functions and must collect data through sensors without the user being aware and without requiring their direct intervention.
(3) They must have the ability to communicate with external devices mediated by the use of any communication technology.
(4) Wearable smart devices must be able to communicate and analyze to achieve their intended functions.

More recently, authors such as Motti (2020) [39] have summarized certain characteristics that wearable devices present or should present, such as: optimizations in size and comfort; wearable accessibility: visual enhancements and voice and gesture recognition software; high degree of configuration, allowing different tools to be grouped into a single device; security and privacy: complying with standards that allow users to protect themselves against attacks and unauthorized access; wearable efficiency through the use of resources and energy; optimized design and architecture; higher levels of personalization according to the needs of each user; integration: technological improvements in adapting to the users’ outfits; interaction: allowing users to focus on their main tasks while interacting with technology; and situational awareness: recognizing the contexts where the interaction occurs and allowing their functions to be adapted to the user’s needs in real time.

Currently, wearable devices offer great opportunities mainly (but not exclusively) in the areas of sports/fitness and health monitoring and care [40]. However, these devices are very useful in other areas such as:

- Work environments: for the prevention of occupational risks [41], control/supervision (with the ethical challenges that this entails) and monitoring of the state of health and well-being of employees [42].
- Marketing and “geographic market intelligence” (GeoMarketing): offering products and services in an innovative and creative way [43].
- Education: allowing to detect the physical information and the interaction results of the students of a school, and then give feedback to the teachers [44]. In addition, they have already been shown to support the diagnosis of disorders such as Attention Deficit Hyperactivity Disorder (ADHD) [27].

As mentioned, the storage and flow of data across multiple sectors is sparking growing concern over legal standards and case law for privacy violations, leading to rethinking the limits on data acquisition and management. Suitable regulatory frameworks must be established in the future [45].

### 2.2. Types of Wearable Technologies and Business Devices

**Figure 1:**
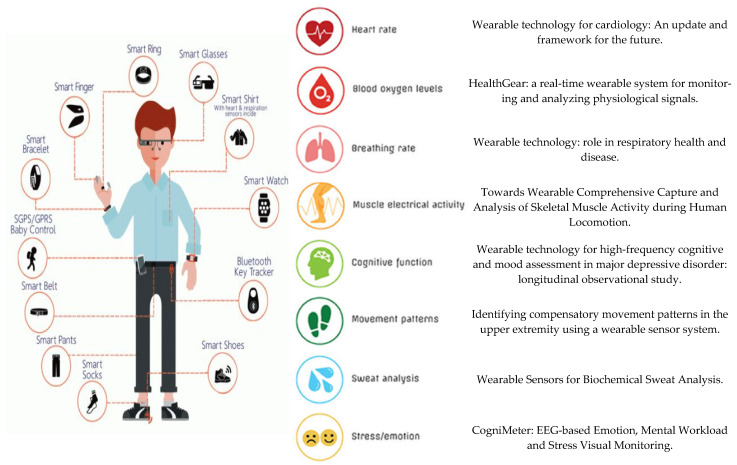
Types of WT and products and examples of applications in healthcare and monitoring [49,50,51,52,53,54,55,56,57,58].

Seneviratne et al. [46] in 2017 carried out a thorough survey and classification of the different types of existing wearable devices and their related products on the market. In this study they presented a subdivision made up of four groups of existing WT; Type 1. Wrist-worns (smart watches and wrist bands); Type 2. Head-mounted devices (smart eyewear, headsets and ear-buds); Type 3. E-Textiles (electronic textiles), smart garments and foot/hand-worn devices); Type 4. E-patch (electronic patch), sensor patches and e-tattoo/e-skin; Type 5. Other (smart jewelry and straps). Figure 1 shows some of the areas on which this study focuses and research is added where different devices for health monitoring have been reviewed and proposed.

### 2.3. Previous Research and Results Achieved

One of the first and most comprehensive bibliometric reviews on the research field of WT was published in 2019 [47]. This paper reviewed the literature published between the years 2000 and 2016. Its results pointed to a rapid growth and importance of the central theme of WT in scientific literature, especially since 2013. The United States led the ranking of most productive countries, followed by China and the United Kingdom. The institution with the highest production rates detected was Keio University. The author with the most publications in the area was Tröster, G., while other authors such as Bonato, P. were also pointed out. However, the main difference with this review is that this study did not focus on the relationship between WT and healthcare.

More recently, in 2021, a systematic review and bibliometric analysis on WT and their interaction with the consumer was published. It highlighted the role of consumer behavior, well-being and decision-making. At the same time, it stressed the usefulness of wearables and big data in marketing studies [48].

Finally, we can point to another bibliometric analysis related to WT and its applications to health carried out in 2018. This study gave a great introduction by reviewing all the literature related to the concepts “Wearable AND Health”. In this review, it was found that the main topic was monitoring systems, along with the appearance of new fields of undoubted value such as mobile technologies and the study of algorithms, an evolution aimed at signal processing [59].

## 3. Methods

### 3.1. Search Strategy

**Figure 2:**
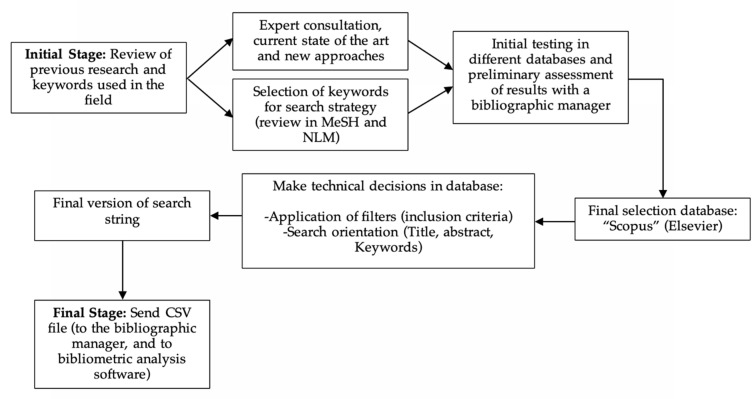
UML diagram of the stages followed in the search strategy.

All the input data for the analysis were retrieved from Scopus, a scientific database. Since its inception, Scopus has been estimated to be the most comprehensive database of the world’s scientific research production in the fields of science, technology, medicine, social sciences, the arts and humanities [60]. For this reason, it was considered that this database would be the most suitable to conduct searches related to the field of WT for monitoring human health and thus avoid the inclusion of unrelated studies. In addition, the searches were carried out in a single day in order to avoid possible bias caused by the database being updated. Different searches were carried out in other complementary databases, finding mostly duplicate records and therefore confirming that the search in Scopus collected the most accurate information according to the objective of this study. This retrieval of input data for further analysis was carried out at the end of 2021. The aim is to analyze full years from 2000 to 2021. A controlled vocabulary was used, and for this, the terms proposed for the search were consulted in the Medical Subject Headings (MeSH) of the National Library of Medicine (NLM). An expert in bibliographic searches advised on current issues related to the research field to develop an adequate bibliographic search strategy. The terms used for the advanced document search are presented in Table 1. The steps followed during the search phase are specified in the UML diagram in Figure 2.

### 3.2. Selection Study and Inclusion Criteria

To determine which of the studies retrieved in the database with this search strategy would be included in this bibliometric analysis, two authors screened the articles independently by reviewing their titles and abstracts. If at least one of the authors decided it was relevant enough to be included in the study, it was assessed in detail during this phase. In the present study, only original articles and reviews were selected for further analysis. Therefore, the first search in the database was refined according to type of study. The included studies had to have been published in the period from 2000 to 2021. This time period was decided based on the recommendations put forward in previous research on the emergence and development of wearable sensors/systems and their impact on biomedical engineering [61,62,63]. No filters were applied either for language or free full-text access. All data retrieved from this search were independently verified by the two researchers (A.-P.A.-R. and A.-J.R.C.). Table 2 outlines the specific criteria followed to screen the studies.

### 3.3. Data Analysis

In order to carry out the bibliometric analysis, it was first necessary to extract bibliometric information from the articles of the Scopus database. The bibliographic record contained information related to various fields, including citations, bibliographical information, abstract & keywords, funding details and other information. All the data were saved in “RIS” and “CSV” file formats. 

For the data analysis, four different research support software programs were used. To visualize patterns and trends in the specialized scientific literature, we used CiteSpace 5.7.R2 (64 bit), under Java Runtime v.8 update 91 (build 1.8.0_91-b15) [64] and VOSviewer version 1.6.15 (Drexel University, Philadelphia, PA, USA) [65]. The statistical analysis was carried out using IBM SPSS Statistics 26 and Microsoft Excel 2016. These software tools have already been widely tested and used in previous bibliometric analyses which have demonstrated their usefulness [66,67,68]. More specifically, the following techniques and software were used for data analysis:

(1) Publication outputs and development trend.
(2) Analysis of countries and keywords: (1) visualization of collaborative networks; (2) history of the burstiness of keywords; (3) diagram pennant of concepts and (4) timeline plot for publications. CiteSpace 5.7.R2 (64 bit) software.
(3) Analysis of authors, co-cited authors, journals and keywords: link strength analysis and cluster views. VOSviewer software version 1.6.15.
(4) Ranking of the most active subject areas: Microsoft Excel 2016.

## 4. Results

### 4.1. Results of the Search and Selection of Studies

**Figure 3:**
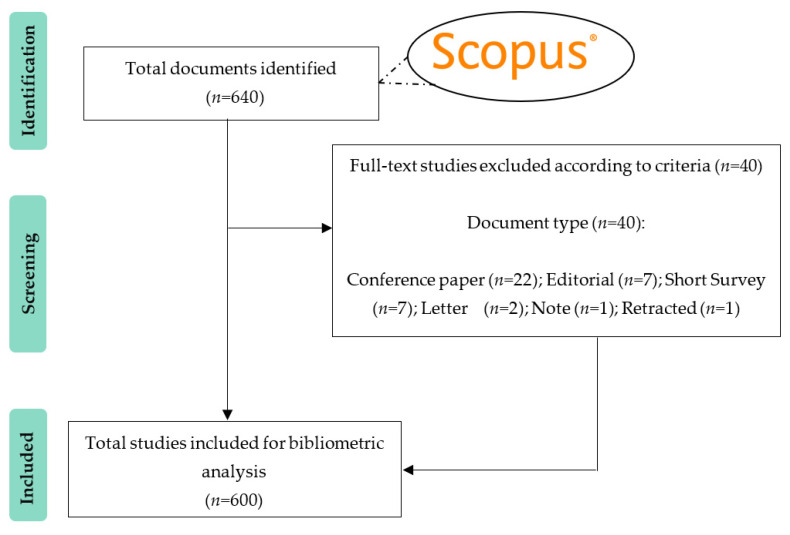
Flowchart of document retrieval from the Scopus database.

A total of 640 documents were retrieved after the advanced literature search in the Scopus database. A blind academic peer review of the title and abstract of each of the documents was conducted. In the event of discrepancies, after reviewing the full text, it was decided not to eliminate any study based on content, since all were related to the subject matter of this bibliometric analysis and were therefore relevant to analyze scientific production on WTH. In the next stage, the studies were filtered by type of document, where a total of 40 studies were eliminated because they did not meet the eligibility criteria proposed for inclusion. It was not necessary to discard any study by publication date since the first two studies found were from 2001. Finally, 600 studies were included for bibliometric analysis. The bibliographic search results and study selection process is shown in the flowchart in Figure 3.

### 4.2. Publication Outputs and Development Trend

Regarding to the SRQ1, a total of 600 documents were retrieved and included for further analysis, of which 491 (81.8%) were articles, while the remaining 109 (18.2%) were reviews. As mentioned, for greater scientific rigor in relation to the type of documents analyzed, only articles and reviews were included in this bibliometric analysis. Compared to articles and reviews, the portion of remaining deleted documents was much lower: Conference paper (*n* = 22; 3.4%); Editorial (*n* = 7; 1%); Short Survey (*n* = 7; 1%); Letter (*n* = 2; 0.3%); Note (*n* = 1; 0.1); Retracted (*n* = 1; 0.1).

The quantitative analysis reveals that research on WT for human healthcare and monitoring has experienced growing interest over the past 20 years, going from the two studies published in 2001 to one hundred and twenty in 2021. 

A subdivision can be seen that covers two key periods in scientific production in this area. The first stage of WT research focused on development and engineering work needed for the design and development of wearable sensors and systems. Then, in the second stage (from 2013 onwards), more recent research has focused on the application of said technology aimed at monitoring health and well-being in general. We point to the review and the special issue headed by Bonato in 2003 [69] as especially representative of the first period: it consisted of five studies, where engineering advances for the design and medical applications of non-intrusive wearable systems that allow doctors to monitor people for long periods were already being discussed. The year 2012 was a key turning point in this area of study, as the growing volume of research made it possible to summarize the concrete contributions and clinical applications of WT, such as in the review by Patel, Park, Bonato, Chan and Rodgers [70], which focused on rehabilitation. Since that time, numerous studies have begun to analyze the future impact and concerns around the use of wearable health technology. In the same way, a large density of experimental and case studies has been carried out for the development and validation of new non-invasive and commercial wearable devices for health monitoring [27,71,72,73].

### 4.3. Analysis of Countries and Institutions

In order to answer to SQR2 an analysis of countries and institutions was performed. Contributions to the literature related to WT applications in healthcare come from academics located in 71 countries. The 10 most active countries that have contributed to the development of scientific production on WTH can be seen in Table 3. The United States leads with 208 published papers, representing 34.7% of the total. Relevant contributions also come from the United Kingdom (68 published documents, 11.3%) and Italy (53 documents, 8.8%). Other countries that have also made significant contributions to the research field are: China (42 articles, 7%), South Korea (38 articles, 6.3%), Spain (35 articles, 5.8%), Australia (34 articles, 5.7%), Germany (33 articles, 5.5%), India (28 articles, 4.7%) and Canada (27 articles, 4.5%). These results are in line with the findings of a similar previous research effort carried out in 2019, which analyzed the literature related to WT for connected health and found that the United States and the United Kingdom were the leading countries in this field of research [74]. Regarding the most active institutions, the University of California, Los Angeles, is the institution with the highest production in WTH (*n* = 19; 3.1%), followed by Harvard Medical School (*n* = 15; 2.5%), the University of Twente (*n* = 10; 1.6%) is tied with the University of Granada (*n* = 10; 1.6%) (Table 3).

**Figure 4:**
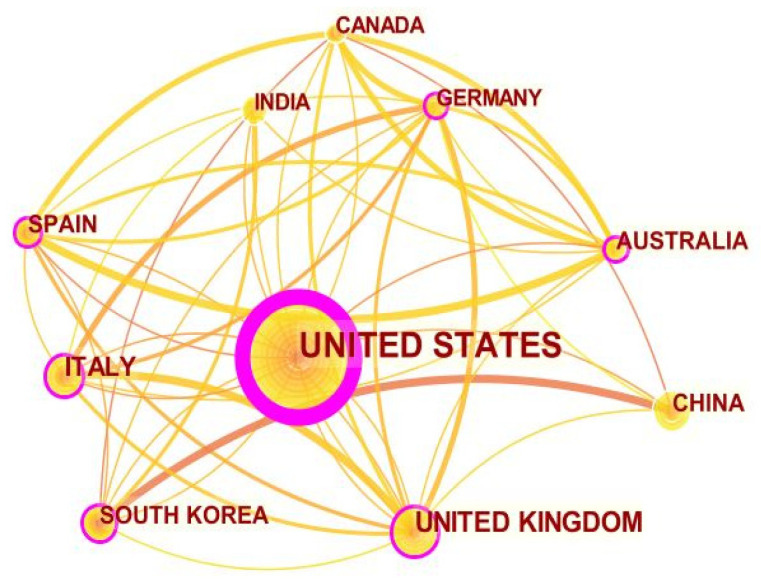
Distribution by country. Larger size and depth of color indicate a greater number of publications and the lines represent their collaboration.

In addition, with the help of the CiteSpace 5.7.R2 (64 bit) software, a network map was made where the connections between countries carrying out research in the field of WTH can be observed. The United States together with the United Kingdom were the leading countries in this regard, as can be seen by the size of the nodes and by the connection lines that converge in these countries (Figure 4).

### 4.4. Journal Analysis

**Figure 5:**
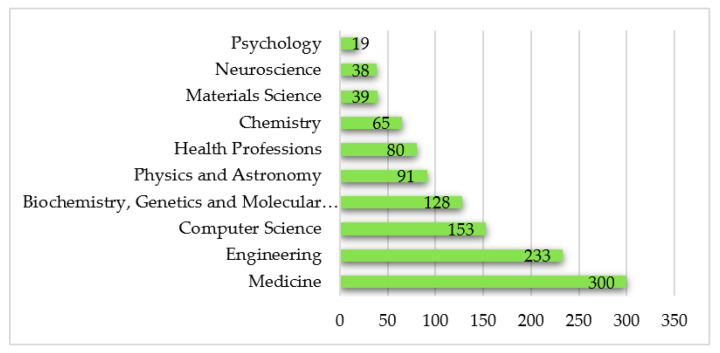
Ranking of top 10 active research areas related to WTH from 2000 to 2021.

To address SQR 3, a journal analysis was conducted. Studies on WTH were published in a total of 344 academic journals. Table 4 shows the top 10 journals indexed in Scopus with the highest number of publications in this field of study. Sensors (Switzerland) leads this ranking with the highest number of published articles (*n* = 51; 8.5%), followed by JMIR mHealth and uHealth (*n* = 31; 5.2%) and by Telemedicine and E Health (*n* = 26; 4.3%). The subject areas with the highest production in relation to WTH are: (1) Medicine, which leads the ranking with 300 articles in this area; (2) Engineering with 233 studies; and (3) Computer science with 153 indexed articles. The top 10 subject areas related to research on WTH are presented in Figure 5. 

**Figure 6:**
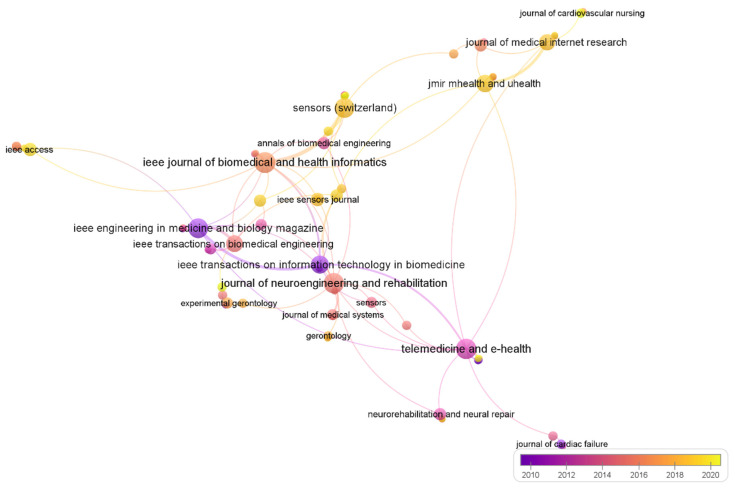
Cluster view of journals with higher link strength obtained with the VOSviewer tool.

Moreover, of the 344 original sources after screening and imposing a limit of one document per source and a minimum of one citation per source, 260 journals met these thresholds. This procedure was performed to calculate the total strength of the citation links between sources. In this sense, the journals with the strongest citation connections were IEE Journal of Biomedical and Health Informatics (16); Telemedicine and E Health (15); and Sensors (Switzerland) (14). At the same time, the graph represents how journals such as JMIR mHealth and uHealth, IEE Sensors Journal or Sensors (Switzerland) have recently been publishing more on WTH than other journals (see Figure 6).

### 4.5. Analysis of Authors and Co-cited Authors

To answer SRQ 4, an analysis of authors and co-cited Authors was carried out. In total, 3142 authors participated in the publication of the 600 documents retrieved, which yields a high average of authors per document (5.2). Within the node distribution, there are two standard attributes used in the VOSviewer program, these are the “links” attribute and the “Total Link Strength” attribute (TLS). In our study, the second attribute is used, which can be defined as the total strength of the links of an item with other items. For example, in the author cocitation network, the attribute value indicates the total strength of a given researcher’s cocitation links with other researchers (see Table 5).

The 10 main authors involved in the research on WTH are listed in Table 5. The authors are ordered in the ranking, not only based on the number of publications, but also based on the total number of citations. Therefore, the greater the total link strength, the better their position will be in the ranking, due to their greater influence. Table 5 shows information on the number of citations of the authors that allows contrasting their importance, from two perspectives, direct citations and co-citations made. Again, the minimum number of documents per author was reduced to one and these authors had to submit at least one citation, leaving a total of 2373 authors who met these requirements. In this sense, the authors with the greatest link strength were Bonato, P. (112); Patel, S. (88) and Rodgers, M. (58), who shares the same position with Park, H. and Chan, L. 

**Figure 7:**
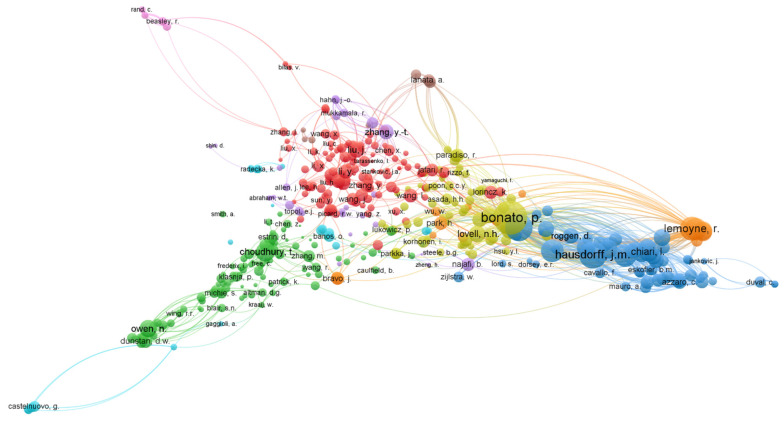
Cluster view of co-cited authors active in the research field of WTH. Each color represents the strongest collaborative networks between authors. The size of the circles is related to greater or lesser co-citation.

The co-citation analysis was also a key criterion in evaluating the degree of contribution to the area. Co-citation makes use of direct citation relationships and is understood as the frequency with which two documents are co-cited by another document, giving them a possible thematic similarity from the citer’s perspective. Figure 7 shows the visualization of the author cocitation network where each node represents a referenced author (unit of analysis) in the scientific production. The size of a node reflects the number of citations an author has received from the analyzed documents. A total of 35,827 authors were co-cited. To improve the visibility of the figure, the minimum number of citations per author had to be set to 10. In this way, 421 authors met the threshold. The top three most co-cited authors according to total link strength were: Bonato, P. (4942); Hausdorff, J.M. (3524); and Patel, S. (3514). (see Figure 7). Referenced authors who are in the visualization close to each other tend to be more strongly related, based on co-citations, than researchers located very far from each other. Therefore, and by way of synthesis, it can be said that at first information is obtained on the most relevant authors in the field based on the number of citations received and subsequently the co-citation links between the most referenced authors in the production are analyzed. scientific.

### 4.6. The Keyword Co-Occurrence Network

To shed light on SRQ 5, the keyword co-occurrence network is studied. Keyword analysis is important since it can offer a more specific vision of the main ideas covered in the papers [75]. With the help of the VOSviewer software, version 1.6.15, a grouped visualization of the keywords with the highest occurrence was generated, which in turn is useful for a better understanding of the research topics of the included studies. Thus, the results show that in the 600 studies analyzed, there are a total of 5816 keywords, of which, when the minimum number of occurrences per word was increased to 10,282 met the criteria.

**Figure 8:**
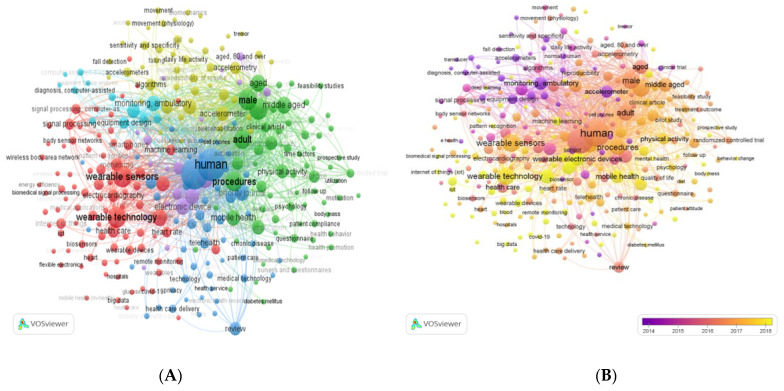
(**A**). Cluster view of frequent keywords in the WTH research field from 2000 to 2021**. Each of the colors within the figure represents a category and keywords that have the same color belong to the same cluster. The keywords “humans” and “article” were removed. “Humans” because it is also presented in the singular “human” and “article” because it is a frequently repeated word with little meaning in this context. The minimum number of occurrences per keyword was kept at 10. (**B**). Distribution of keywords based on year of appearance. ** Data retrieval for this research was carried out at the end of the year 2021.

The keyword co-occurrence network map shows the main themes within the research field, with a visual representation of the terms that appear most frequently: human (379 times); telemedicine (342 times); wearable sensors (176 times); wearable technology (169 times); wearable electronic devices (97 times); ambulatory monitoring (78 times); and mobile application (77 times) (See Figure 7). Figure 8B represents the distribution and frequency of these keywords according to when they were published. As can be seen, ambulatory monitoring was a frequent research topic until 2014; however, the trend shows that the study of mHealth and WT has been prioritized more recently.

**Figure 9:**
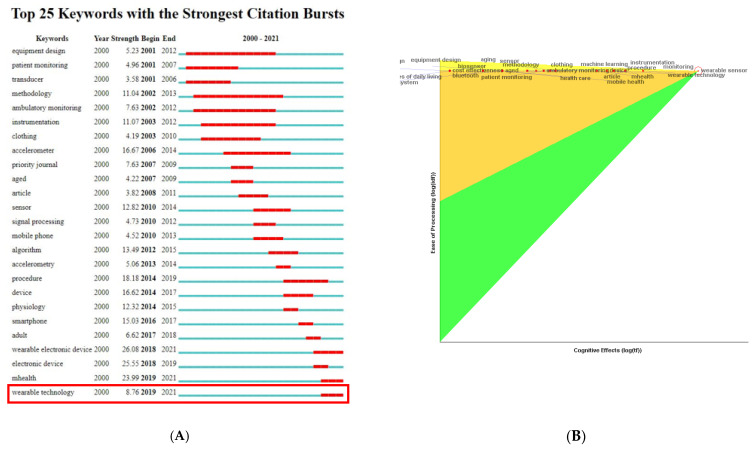
(**A**) Visualization of the history of the burstiness of keywords in publications on WTH (2000–2021 **). Wearable Technology, for example, has the longest burst period from 2019 to 2021 **. (**B**) Pennant chart of concepts related to the term “wearable technology”. ** Data retrieval for this research was carried out at the end of the year 2021.

This information is also corroborated with the use of the CiteSpace software, version 5.7.R2. As can be seen in Figure 9A, there was a burst in the frequency of appearance of the concept of “ambulatory monitoring” between 2002 and 2012. However, according to the keyword analysis carried out with VOSviewer, version 1.6.15, the most recent studies (2019–2021**. (** Data retrieval for this research was carried out at the end of the year 2021)) focus on the analysis of WT. Moreover, the concept of WT appears related, as presented in the pennant chart, with terms related to health monitoring such as: (1) wearable sensor; (2) monitoring; (3) mHealth; or (4) health care (See Figure 9B).

The largest group within the classified terms (*n* = 6) appears related to the concept of sedentary lifestyle (#0), followed by pulse oximetry (#1), transducer (#2), biomedical engineering (#3), wearable sensor (#4) and Internet (#5). The main terms related to these groups appear in Table 6 below. Many terms that make up these general groups are clustered in the period between 2001–2007, while concepts related to more recent terms (#4, #5) show less interaction with other keywords. 

## 5. Discussion

Due to the increase in scientific production in the research field of wearables aimed at monitoring and healthcare, it was considered relevant to present a bibliometric study that would detect the trends and evolution of this study area. The aim of the present bibliometric analysis was to map the scientific advances produced in WTH, identify new challenges and put forward some proposals to address them.

Regarding to the SRQ1, in our study, as in other previous works [47], we detected that there has been a notable increase in the literature on WTH since the year 2013. Until then, the main contributions to the field were made by Bonato, P. [76,77], especially with the study “A Review of Wearable Sensors and Systems with Application in Rehabilitation” [70] and the coordination of the special issue “Health Informatics and Personalized Medicine” [78]. It would not be an overstatement to praise both the review and the subsequent publication of the full special issue for laying solid foundations for the engineering design of portable applications in healthcare. 

In the second period of scientific production from 2013 onwards, Patel, S.’s contributions to the field stand out particularly. His studies are mainly related to the contributions of WT and inertial sensors for monitoring and rehabilitation [79,80,81,82,83,84,85,86]. Inertial sensors present different fields of application within biomedicine such as: (1) gait analysis [87]; (2) orthopedics and rehabilitation [88] and in (3) measurement of sports performance [89].

In relation to SRQ2, the results obtained around the most active institutions in the field of WTH, it is worth highlighting the University of California, Los Angeles. This institution boasts one of the most competitive research laboratories in the world. The Billi Research Lab [90] carries out research mainly focused on the analysis of possibilities and development of:

(1) Orthopedic biology
(2) Wearable technologies
(3) 3D printing
(4) Smart and functional materials
(5) Smart textiles

In connection with the SQR3, it seems relevant to discuss the evolution that has been taking place around the academic journals selected for the publication of manuscripts. Considering the two publication periods that we established in this manuscript—the first period (until 2013) and the second period (from 2013 onwards)—we have observed that researchers worldwide at first opted for medical engineering journals (IEEE Engineering in Medicine and Biology Magazine and IEEE Transactions on Biomedical Engineering) while more recently pivoting towards sensor and mHealth journals (Sensors (Switzerland) and JMIR mHealth and uHealth) for publications on this topic. 

Regarding to SQR4, the authors with the greatest link strength were Bonato, P. (112); Patel, S. (88) and Rodgers, M. (58), who shares the same position with Park, H. and Chan, L. The top three most co-cited authors according to total link strength were: Bonato, P. (4942); Hausdorff, J.M. (3524); and Patel, S. (3514).

In answer to SQR5, the keyword analysis reinforces these notions about the evolution of the field of study of WTH, with terms such as “wearable sensors”, “wearable technology”, “telehealth”, “computer-assisted diagnosis”, “accelerometers”, “mobile health”, “physical activity” or “gyroscope” being the most frequent. These data point to the growth of wearables as a leading category within research related to the Internet of Things. This analysis also allows us to better understand the sensor systems studied. Within the range of available sensors, the data seem to point to more research emphasizing the usefulness of accelerometers and gyroscopes. However, we must add the caveat that merely the detection of the most researched sensor categories is not enough to confirm this—more detailed analyses on the challenges that wearables face in the detection and processing of data, such as the power consumption of portable devices [91], would be required.

In the pennant chart, we show the main concepts that appeared in connection with WT. Among these we can highlight the relationship between this concept and wearable sensors, giving us a glimpse into the most recent research trends. Traditional portable devices present various difficulties, including discomfort due to prolonged use and insufficient precision. In recent years, there has been a greater advocacy for obtaining macro-health data using smart clothing, mediated by the Internet of Things. This would greatly facilitate the discreet collection of physiological indicators of the human body [92,93,94,95,96].

We consider it relevant to make a special mention of the growing interest in research groups around the world in relation to the design and development of new chemical and biochemical sensors [97]. This is evidenced by the increasing number of international scientific articles published in this area. Currently, institutions such as the National Institute of Biomedical Imaging and Bioengineering (NIBIB) [98] are funding research on:

- Circulating nanosensors for continuous drug monitoring.
- Smart textiles for the prevention of deep vein thrombosis.
- Inexpensive genetic sensors for zinc deficiency.

The combination of wearable (bio)chemical sensors with physical sensors used in wearable technologies is becoming an attractive alternative to the bulky and expensive analytical instruments used in the medical sector [99]. As the capacity and performance of non-invasive biosensors improves, it becomes more feasible for wearable devices with physical sensors to also incorporate this type of sensor in their design [100].

The era of healthcare 4.0 has arrived full of challenges. At a global level, it could be said that these challenges focus on improving Internet of Medical Things (IoMT) devices in terms of data collection, integration and interpretation [101], as well as the creation of interfaces capable of supporting a more permanent, objective and holistic monitoring [102]. New advances are being made, for example, with the design and development of IoT middleware for the integration of heterogeneous WTs with healthcare platforms [103]. 

More precisely, the most recent literature points to certain limitations in portable health devices (HWDs), such as, for example, the distortion and noise collected by sensors that interact with the epidermis due to the constant movements of the body and the hair, altering its qualities for making diagnoses. Similarly, HWDs still exhibit limited integration in the presence of biological samples such as blood, urine or saliva. Other challenges that will need to be addressed in the immediate future include the management of personal information, privacy and security [104]; energy consumption (where advances are being made from the field of self-powered sensors such as the piezoelectric nanogenerator, PENG, and the triboelectric nanogenerator, TENG); more research being required for their applicability in psychological diseases (an under-researched area to date) [105]; and finally, from a materials perspective, the development of breathable, flexible and elastic materials remains a major challenge [106].

### 5.1. Limitations 

The present bibliometric analysis is not without its limitations. The main ones are related to the heterogeneity found in the publications within the field of WTH. The specificity of the search chain proposed led to the collection of documents focusing specifically on the role of wearable technologies in healthcare and monitoring. Although this was really the goal of our paper, there are limitations to this approach.

For example, when pointing to the most prominent authors in the field, the first author in our ranking is Bonato, P. with only 9 studies. Although Bonato, P. has made important and massive contributions to the field of wearable technologies, by limiting our search terms to concepts related to healthcare and monitoring, the contributions retrieved are diminished. This can be considered as a drawback as it limits the practical utility of mapping the data, while narrowing down the overall objective of our paper. Similarly, it may also happen that relevant authors or journals in the generic field of study of WTH have been left out of the analysis due to slight variations in terminology or in the lines of research. Thus, topics of great interest may be lost in this analysis, because the concepts present in the search strategy did not interact with those present in the studies. In future bibliometric research we will start with a broader approach and design search strategies with a larger scope to retrieve more evidence and gather more contributions in the field of study. In the same way, in future works we expect to be able use information review and metasynthesis methods to analyze the contributions of portable (bio)chemical sensors used together with physical sensors, as well as the role played by diagnostic support systems for various pathologies, making specific classifications of these diseases and the systems used to date for their management.

The same occurs for institutions, where the organization with the largest number of publications is the University of California, Los Angeles, with only 19 studies, or with journals, showing Sensors as the journal with the highest number of published studies with 51 articles. However, as we said before, this in turn has helped us to focus more precisely on the subject matter at hand: the applications of WTH.

### 5.2. Implications

Wearable technologies measured by the use of sensors are being increasingly marketed. This comes as no surprise, given their benefits for physical monitoring—requiring low intervention from the user and professionals—and their low cost. In this context, research pointing to the current trends in this field of study, providing a glimpse of the possibilities of these devices as well as the improvements that are still necessary, becomes key. One of the contributions of this study, in addition to mapping the bibliometric information on WTH, has been to introduce and discuss the implications of wearable technologies in the field of health care. In this regard, we present some of the most relevant contributions of these devices to human health:

Early diagnosis of symptoms, thanks to obtaining more precise parameters.

Personalization of software to the user according to their needs.

Patient surveillance, control and monitoring, offering the possibility of monitoring patient evolution in real time.

Adherence to treatments: “biofeedback” that encourages the patient to adhere to their treatment and to have greater control over it.

Registration of information and connectivity: data storage in real time and more precise analysis of the same. This in turn allows a more comprehensive and accurate medical history to be generated.

Savings in healthcare costs: remote care through devices saves time, material and personnel costs.

The main stakeholders or target groups of this research will be mainly hospital administration managers, clinical medicine professionals, academic professionals, experts and engineers from the commercial device industry, patients, people with disabilities, medical conditions or chronic conditions, and specialized researchers in the fields of health, systems engineering and more specifically biomedical engineering. However, it may also be of interest to general business managers, wearable garment designers, marketing experts, athletes, coaches and society in general.

## 6. Conclusions

In this work, a bibliometric analysis of the scientific production on WTH using the Scopus database has been presented. This study has discerned the great thematic heterogeneity that exists within the field of study of wearable technologies. However, this analysis has focused on wearable technology in healthcare, a field in which we have seen that there are yet to be authors or institutions with a large scientific production. These low production rates appear to be due to the relatively recent appearance and use of these devices in healthcare.

The results of our study show some of the existing gaps within the field of WTH, such as the need to improve the energy performance of devices and to open new avenues for interdisciplinary cooperation between healthcare professionals and sensor engineering experts. Energy efficiency needs to be improved in order to increase the lifetime of these devices and to ensure alignment with SDG 7 (affordable and non-polluting energy). There is also a need to design wearable devices with a long lifetime, and search for methods to promote a circular economy (SDG 11 and 12).

Taking into account the findings that have been presented in this article, future research could analyze the operation of these devices in detail through their use in clinical trials with diverse populations such as: elderly people, people with impairments, conditions, syndromes or disabilities, among others. Expanding the field of study to the use of smart clothing specifically shows particular potential. Furthermore, these studies must be accompanied by strategies that provide guidance for the reduction of production costs of the systems so that they are made accessible to any household and more and more people may benefit.

The results of this work can be submitted to knowledge transfer processes with companies and public/private institutions engaged in the design, development and management of wearable systems for monitoring health and well-being. Dissemination strategies, training, collaboration contracts, transfer agreements, research contracts and consulting agreements can be used for this purpose.

## Figures and Tables

**Table 1:** Bibliographic search strategy.

Search for Keywords
TITLE-ABS-KEY (((e-health OR m-health OR “mobile health” OR u-health OR telemedicine) AND (imu OR gyroscope OR accelerometer OR “wearable sensor” OR “wearable technology”))) AND (monitoring)
Filters applied
(LIMIT-TO (DOCTYPE, “ar”) OR LIMIT-TO (DOCTYPE, “re”))

**Table 2:** Eligibility criteria.

Criteria	Values
Database	Scopus (Elsevier)
Document types	Original articles and reviews
Document contents	Empirical and theoretical studies related to the aplplications of wearable technology in healthcare (WTH)
Date of publication	From 2000 to 2021

**Table 3:** Top 10 active countries and institutions on WTH research from 2000 to 2021 **.

Rank	Country	Frequency (%) *n* = 600	Institution	Frequency (%) *n* = 600
1st	United States	208 (34.7)	University of California, Los Angeles	19 (3.1)
2nd	United Kingdom	68 (11.3)	Harvard Medical School	15 (2.5)
3rd	Italy	53 (8.8)	University of Twente	10 (1.6)
4th	China	42 (7)	Universidad de Granada	10 (1.6)
5th	South Korea	38 (6.3)	David Geffen School of Medicine at UCLA	9 (1.5)
6th	Spain	35 (5.8)	Imperial College London	9 (1.5)
7th	Australia	34 (5.7)	University of Oxford	9 (1.5)
8th	Germany	33 (5.5)	Université McGill	8 (1.4)
9th	India	28 (4.7)	IRCCS Istituto Auxologio Italiano	8 (1.4)
10th	Canada	27 (4.5)	University of California, San Francisco	8 (1.4)
	Total	566 (94.3)	Total	105 (17.5)
	Others	34 (5.7)	Others	495 (82.5)

Note: For practical purposes, the countries and institutions are ranked in order, although, as can be seen, there are institutions with the same number of publications. ** Data retrieval for this research was carried out at the end of the year 2021.

**Table 4:** The 10 most active journals in the field of WTH.

Rank	Journal	Records, (%) *n* = 600	Citations	TLS	Country	Subject Area (Category)
1st	Sensors (Switzerland)	51 (8.5)	1461	14	Switzerland	Biochemistry, Genetics and Molecular Biology (Biochemistry); Chemistry (Analytical Chemistry); Computer Science (Information Systems); Engineering (Electrical and Electronic Engineering); Medicine (Medicine (miscellaneous); Physics and Astronomy (Atomic and Molecular Physics, and Optics; Instrumentation).
2nd	JMIR mHealth and uHealth	31 (5.2)	446	10	Canada	Medicine (Health Informatics).
3rd	Telemedicine and E Health	26 (4.3)	736	15	United States	Health Professions (Health Information Management); Medicine (Health Informatics; Medicine (miscellaneous)).
4th	IEEE Journal of Biomedical and Health Informatics	21 (3.5)	902	16	United States	Biochemistry, Genetics and Molecular Biology (Biotechnology); Computer Science (Computer Science Applications); Engineering (Electrical and Electronic Engineering); Health Professions (Health Information Management).
5th	Journal of Medical Internet Research	18 (3)	380	8	Canada	Medicine (Health Informatics).
6th	IEEE Access	12 (2)	146	5	United States	Computer Science (Computer Science (miscellaneous)); Engineering (Engineering (miscellaneous)); Materials Science (Materials Science (miscellaneous)).
7th	IEEE Sensor Journal	11 (1.8)	180	5	United States	Engineering (Electrical and Electronic Engineering); Physics and Astronomy (Instrumentation).
8th	IEEE Transactions on Biomedical Engineering	11 (1.8)	465	9	United States	Engineering (Biomedical Engineering).
9th	IEEE Transactions on Information Technology in Biomedicine	9 (1.5)	1517	11	United States	Computer Science (Interdisciplinary Applications); Medical Informatics; Mathematical & Computational Biology; Computer Science (Information Systems).
10th	Journal of Medical Systems Total Others	9 (1.5) 199 (33.2) 401 (66.8)	296	3	United States	Computer Science (Information Systems); Health Professions (Health Information Management); Medicine (Health Informatics; Medicine (miscellaneous)).

Note: For practical purposes, the journals are ordered according to number of publications on WTH, although as can be seen there are journals with the same results. Abbreviations: TLS = Total Link Strength; SA: Subject Area.

**Table 5:** The top 10 most influential authors and co-cited authors on WTH.

Rank	Author	Records	Citations	TLS	Co-Cited Authors	Citations	TLS
1st	Bonato, P.	9	1646	112	Bonato, P.	86	4942
2nd	Patel, S.	4	1304	88	Hausdorff, J.M.	52	3524
3rd	Rodgers, M.	1	1126	58	Patel, S.	63	3514
4th	Park, H.	1	1126	58	Lemoyne, R.	31	2760
5th	Chan, L.	1	1126	58	Giladi, N.	31	2597
6th	Parisi, F.	2	40	52	Aminian, K.	65	2538
7th	Mauro, A.	2	40	52	Mastroianni, T.	27	2440
8th	Ferrari, G.	2	40	52	Troster, G.	43	2288
9th	Cimolin, V.	2	40	52	Chiari, L.	26	2280
10th	Azzaro, C.	2	40	52	Horak, F.B.	20	1964

Note: For practical purposes, the authors who have published the most studies and have the highest average number of citations on WTH have been ordered, although as you can see there are different authors with the same results. Abbreviations: TLS = Total Link Strength.

**Table 6:** Six research clusters in the field of WTH.

Cluster, Ranked Terms	Top Terms
#0	system; information; network; wireless; standard heart; blood; electronic; diagnosis; pressure
#1	algorithm; rate; oximetry; respiratory; pulse care; agent; walking; test; heart
#2	heart rate; risk; hospital; textile sleep; mental; dementia; prospective; caregiver
#3	biomonitoring; e textile; electroactive polymers (EAPs); electronic textile; polymer actuator; polymer battery; polymer electronics; polymer sensor; rehabilitation and telemedicine; smart textile; wearable sensor; actuator; biomedical engineering; biosensor; elastomer; human computer interaction; human rehabilitation equipment; intelligent material; patient monitoring; computing methodology; diagnosis, computer-assisted; electrochemistry; equipment design; monitoring, ambulatory; polymer; telemedicine; telemetry; textile; transducer
#4	wireless; signal; internet; electrocardiography; movement aged; male; sleep; textile
#5	general practitioner/GP; Internet; telemedicine; biological monitoring; environment; human; Internet of Things; remote sensing; technology; telecommunication; United States; human; image processing, computer-assisted; mountaineering

## Data Availability

All relevant data are within the paper, and those are available at the corresponding author. For a better visualization of the key figures, you can visit the following link: https://github.com/AntonioAlbin-dev/wearable_healthcare (accessed on 27 October 2022).
